# Bio-Cooperative Approach for the Human-in-the-Loop Control of an End-Effector Rehabilitation Robot

**DOI:** 10.3389/fnbot.2018.00067

**Published:** 2018-10-11

**Authors:** Francesco Scotto di Luzio, Davide Simonetti, Francesca Cordella, Sandra Miccinilli, Silvia Sterzi, Francesco Draicchio, Loredana Zollo

**Affiliations:** ^1^Research Unit of Biomedical Robotics and Biomicrosystems, Università Campus Bio-Medico di Roma, Rome, Italy; ^2^Unit of Physical and Rehabilitation Medicine, Università Campus Bio-Medico di Roma, Rome, Italy; ^3^INAIL, Department of Occupational & Environmental Medicine, Monte Porzio Catone, Rome, Italy

**Keywords:** upper limb robot-aided rehabilitation, arm-gravity support, human-in-the-loop, biocooperative control, muscle activation

## Abstract

The design of patient-tailored rehabilitative protocols represents one of the crucial factors that influence motor recovery mechanisms, such as neuroplasticity. This approach, including the patient in the control loop and characterized by a control strategy adaptable to the user's requirements, is expected to significantly improve functional recovery in robot-aided rehabilitation. In this paper, a novel 3D bio-cooperative robotic platform is developed. A new arm-weight support system is included into an operational robotic platform for 3D upper limb robot-aided rehabilitation. The robotic platform is capable of adapting therapy characteristics to specific patient needs, thanks to biomechanical and physiological measurements, and thus closing the subject in the control loop. The level of arm-weight support and the level of the assistance provided by the end-effector robot are varied on the basis of muscular fatigue and biomechanical indicators. An assistance-as-needed approach is applied to provide the appropriate amount of assistance. The proposed platform has been experimentally validated on 10 healthy subjects; they performed 3D point-to-point tasks in two different conditions, i.e., with and without assistance-as-needed. The results have demonstrated the capability of the proposed system to properly adapt to real needs of the patients. Moreover, the provided assistance was shown to reduce the muscular fatigue without negatively influencing motion execution.

## 1. Introduction

Stroke survivors are often left with severe impairments and huge limitations in arm motor abilities that may compromise many common activities.

In such a context, robot-aided neuro-rehabilitation has been globally acknowledged as an effective therapeutic approach for motor recovery after stroke, especially for the upper extremities. Rehabilitation robots are used for improving the therapy outcome and measure the improvements with objective indicators.

While, in the past, emphasis has been put mostly on planar exercises (Kwakkel et al., 2008), recently the importance of performing activities in the 3D space has been pointed out (Klamroth-Marganska et al., 2014). Thanks to rehabilitation exercises in the 3D space, the impaired subjects can regain functional abilities to perform activities of daily living (ADL). In the field of rehabilitation robotics, bio-cooperative systems represent a novel generation of robotic platforms that promote a mutual human-robot interaction based on multimodal interfaces (Simonetti et al., 2017). Data coming from biomechanical, physiological, and psychological measurements, as well as data related to the user's intention and the environmental factors may contribute to provide a continuous feedback on patients' global conditions (Riener and Munih, 2010), and therefore to realize a personalized therapy. To provide the correct level of assistance, tuned on the patient's needs and performance, it is paramount to encourage subject's voluntary participation, promote neural plasticity, increase the potential for recovery of motor coordination, and realize a more effective training (Pehlivan et al., 2016) based on the patient's needs. A human-in-the-loop approach represents a winning strategy to try reaching this goal, being based on the inclusion of the human being in the robot control loop. This tight interaction between humans and robots is based on the adaptation of the robot behavior to the subject needs, thanks to the continuous monitoring of the patient's state and the active inclusion of the patient in the robot control loop by means of different types of feedback (i.e., visual, audio, haptic, etc.).

As a result, robotic assistance can be dynamically changed on the basis of the subject's needs measured by multisensory monitoring systems (Mihelj et al., 2007). This approach is called “assistance-as-needed.” In Riener et al. (2009), biomechanical and psychophysiological measurements are used for including the human in the loop; in Guerrero et al. (2010), psychophysiological feedback is used to develop a human-centered approach method aimed to customize therapy on patient requirements and state, without affecting stress level and health. In Rodriguez-Guerrero et al. (2017), psychophysiological measurements are used for improving the challenge/skill ratio experienced by the user during the interaction with a multimodal interface in a cooperative scenario. Position error is used in Krebs et al. (2003) to measure motion accuracy and adjust the level of robot assistance accordingly.

Robot-aided rehabilitation systems often adoptelectromyographic (sEMG) signals. This type of data represents the most simple and intuitive way to trigger the support provided by the robot. EMG-based robot adaptation is adopted if the subject is able to contract the muscles, but is not able to perform a complete movement (Simonetti et al., 2017). In this case sEMG signals can be used to trigger the movement performed by the robot, to control robot movements through muscles contraction, or to vary the value of the assistance provided by the robot, as in Song et al. (2013). Other online approaches vary the level of assistance based on the obtained performance (Marchal-Crespo and Reinkensmeyer, 2009) or the application scenario (Zollo et al., 2001; Formica et al., 2005).

One of the main drawbacks of these systems is that the gravity effect due to the weight of the upper limb is often not considered.

Supporting the weight of the patient's arm is a key point in post-stroke rehabilitation, since it limits the unhealthy effects of abnormal muscular patterns (Johnson, 2006; Prange et al., 2015). In Amirabdollahian et al. (2007) it was demonstrated that a gravity compensation strategy based on sling suspension led to an improvement of arm function of stroke patients after 9 weeks of training. Therefore, the sole application of gravity compensation might be a valuable strategy to foster functional improvement in post stroke subjects.

Exoskeleton robots can provide compensation of the arm weight and apply forces to several segments of the arm to help the subject performing the desired task (Lauretti et al., 2018). The main drawbacks of these systems are the reduced adaptability to subject's different anthropometry, the passive gravity compensation, the significant amount of time needed for setting-up the device for a particular patient and therefore the complexity of the control algorithms (Maciejasz et al., 2014).

End-effector-based devices can overcome the limitations of exoskeleton robots related to anthropometry adaptability, facility in setting-up and control algorithm complexity.

The main drawback of these systems is that the provided arm support depends only on spatial limb configuration, since gravity torque is highly coupled with limb dynamics. Therefore, subjects voluntary participation and their muscular activation patterns might be affected.

Ideally, arm gravity compensation should guarantee the required assistance without altering users' physiological muscular activation patterns and their voluntary participation.

This paper aims at proposing a novel bio-cooperative platform for robot-aided 3D upper-limb rehabilitation. It is composed of an end-effector robot and an arm-weight support able to overcome the limitations pointed out in the literature. The patient is included in the control loop by continuously monitoring his/her state, extracting objective biomechanical and electromyographic indicators and, consequently, adapting the level of assistance provided by the robotic platform. In the last few years, researchers developed innovative methods to detect the level of muscular fatigue of the subject via sEMG signals (González-Izal et al., 2012). In particular, in Dimitrov et al. (2006), a simple and efficient algorithm to extract fatigue level during dynamic contractions is presented. Muscular fatigue represents an important parameter to assess patient state and adapt the level of support provided by a robotic platform in order to ensure the correct level of assistance. Therefore, user performance and muscular fatigue are taken into account to fit the level of assistance on the patient specific characteristics guaranteeing a patient-tailored therapy together with an assistance-as-needed approach.

During 3D rehabilitation with an end-effector robot, the user can assume incorrect postures during the execution of the task if he/she cannot autonomously support the arm weight, as in the case of impaired people. The introduction of the arm-weight support wants to face this issue by sustaining the patient's limb, according to his/her muscular fatigue level. The complete platform composed of the robotic arm and the arm-weight support is designed for achieving a two-fold purpose: to properly adapt the level of assistance to the patient's specific needs (through the end-effector robot), and to online assess the patient's muscular fatigue and avoid incorrect posture (through the arm-weight support).

A preliminary evaluation of the effects of the proposed platform on healthy subjects is performed in order to (i) give a complete picture of the subject's state and ensure his/her complete integration inside the control loop, (ii) demonstrate that the proposed platform does not negatively affect motor execution and muscular activation patterns. Therefore, muscular activity of the anti-gravity muscles and biomechanical indicators were extracted from 10 healthy subjects during the execution of state-of-the-art 3D point-to-point movements in two different conditions, i.e., with and without assistance provided by the end-effector robot and by the arm-gravity support. The execution of the task without assistance (i.e., in a condition where the healthy subject is not “constrained” by the assistance) represents the best ground truth for evaluating possible effects of the platform on the subject's motor execution and muscular activation patterns.

A comparative analysis between the two different conditions was performed by means of biomechanical and electromyographic indicators to evaluate effects on movement kinematics and muscular activation patterns. The same indicators were also used to develop a bio-cooperative control strategy in order to adjust robotic assistance on the basis of the patient's state. Furthermore, the kinematics of the arm movement is preserved in all arm-weight support conditions while, as suggested by previous studies (Prange et al., 2009), other weight compensation strategies may affect the muscular activation patterns of the upper-limb muscles used for 3D arm reaching movements.

The paper is organized as follows. In section 2 the bio-cooperative robotic platform, the experimental setup and protocol are presented. Experimental results are illustrated and discussed in sections 3 and 4, respectively. Finally, conclusions and future work are reported in section 5.

## 2. Materials and methods

The components of the proposed bio-cooperative system for robot-aided 3D upper limb rehabilitation are described in the following.

### 2.1. An overview of the proposed robotic platform

The proposed robotic platform is composed of a 7-DoFs anthropomorphic robot arm (i.e., the Kuka Light Weight Robot 4+ Bischoff et al., 2010), a purposely developed motorized arm-weight support system and a multimodal interface. It includes an adaptive interaction control for the on-line evaluation of patient performance. The level of assistance is modified by adaptively and dynamically adjusting stiffness and arm-gravity support.

**Figure 1:**
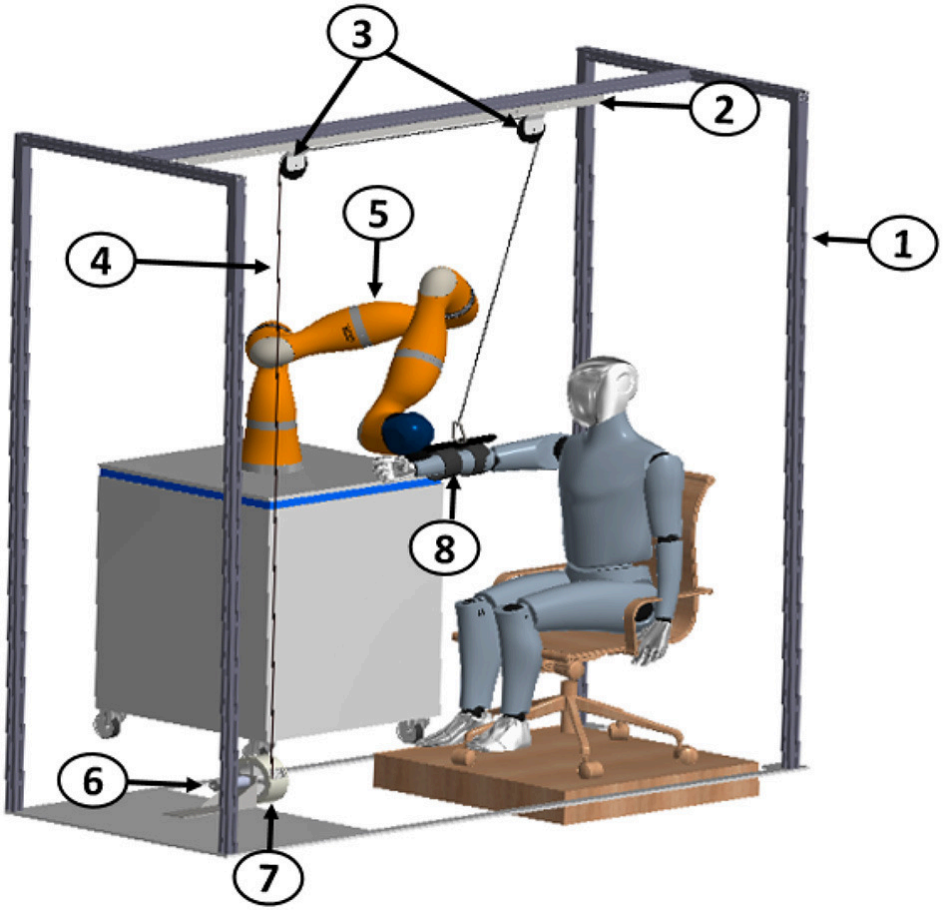
Mechanical structure of the adaptive arm-gravity support system: 1 Frame, 2 Support bar, 3 Pulleys, 4 Cable, 5 7-DoF robot arm Kuka LWR4+, 6 Maxon EC-max 40 motor, 7 Encoder, 8 Ergonomic backing for the arm.

The overall system, presented in Figure 1, is devised as an end-effector machine that, interacting with the patient at the end-effector, offers assistance during point-to-point movements both in 2D and 3D space, as well as in activities of daily living (ADLs). Moreover, an additional mechatronic arm-weight support system has been developed. To this purpose, an adaptive level of support is provided by compensating the gravity force acting on the arm depending on both the subject's performance and the arm configuration in the space. In Figure 1 the arm-weight support is shown together with the whole platform that records hand Cartesian position and provides the elbow Cartesian position to be tracked during the execution of the task. The pullies are used only for the arm-gravity support. The structure around the robot arm makes the system modular and the arm-weight support easily usable with other systems for upper-limb rehabilitation.

The overall robotic platform is based on an adaptive strategy that allows personalizing the therapy including the human-in-the-loop, and assisting the patient as needed in performing rehabilitation treatment. For further promoting patient motivation and engagement, the selected task is reproduced and updated according to the patient behavior in a virtual reality environment (VR) developed in Matlab. VR is composed of a virtual limb that is able to move along 3D selected directions (as described in section 2.3), in order to reach the assigned targets, based on robot end-effector (i.e., subject hand) position.

**Figure 2:**
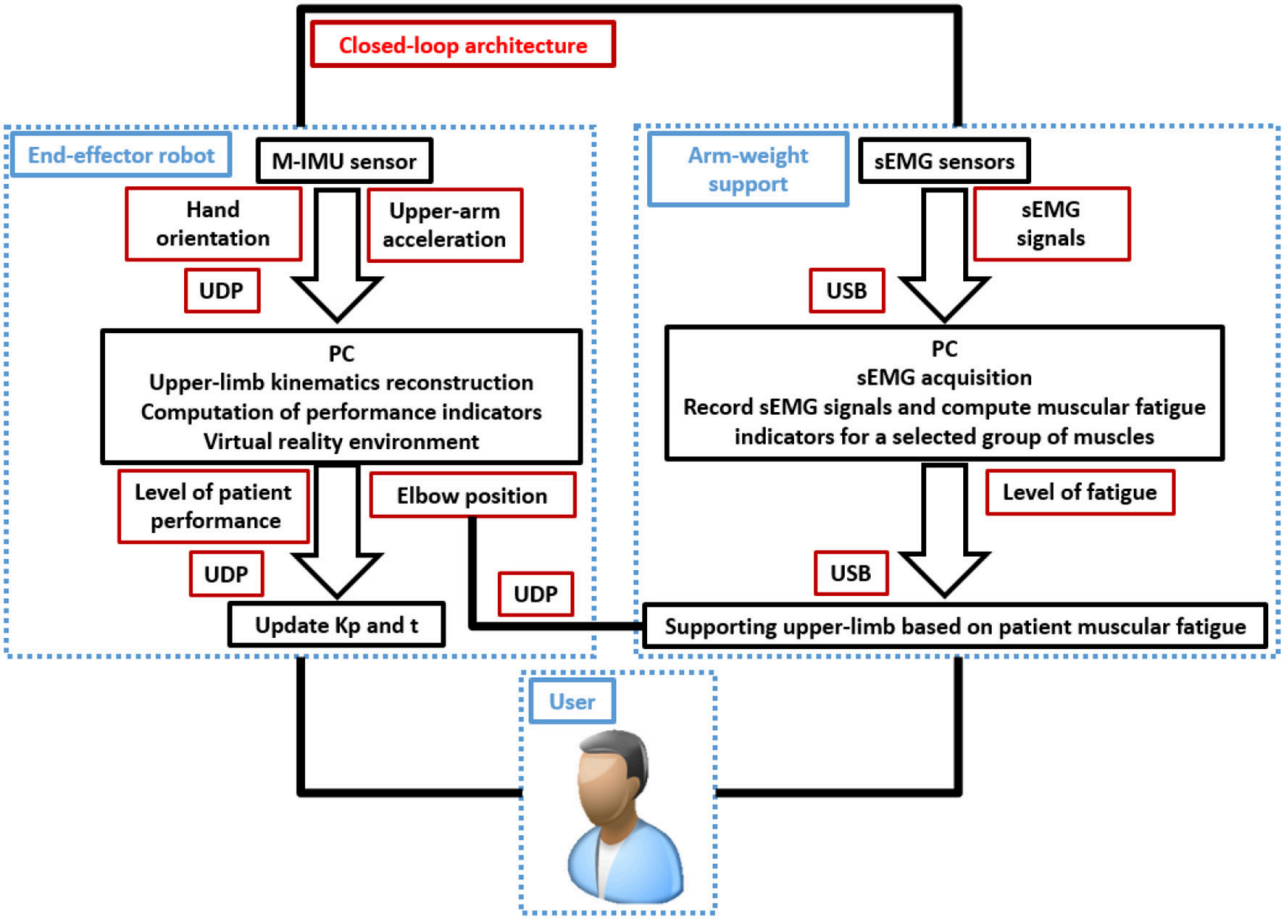
Block scheme of the proposed closed-loop architecture.

During the exercise execution, the subject's wrist is attached to the robot arm end-effector that provides the subject with assistance-as-needed during the execution of a predefined trajectory. The encoders at the joint and the robot forward kinematics provide hand 3D trajectory. The robotic platform is composed of two independent modules (i.e., end-effector robot and arm-weight support) that communicate through USB and UDP protocols (Figure 2).

The proposed platform is able to provide the correct level of assistance thanks to the close interaction between end-effector robot and arm-weight support, as shown in Figure 2. More in detail, the correct level of assistance is assured through:

- the arm-weight support, by increasing or decreasing the weight of the arm felt by the subject. The level of the arm-weight support is evaluated through the level of muscular fatigue, measured by sEMG, as described in section 2.2.1
- the robotic arm, by helping the subject to complete the required task. This level of assistance depends on biomechanical indicators, as described in section 2.2.3.

The multimodal interface is characterized by the following sources of information, suitably merged together to provide a picture of the patient condition: (i) robot sensors for determining hand pose, (ii) a magneto-inertial unit (M-IMU) for reconstructing the user upper-extremity joint motion, and (iii) electromyographic (EMG) electrodes for recording muscular activity and selecting the correct amount of arm-gravity compensation. M-IMU is positioned on subject upper arm, while EMG signals are recorded from the upper trapezius (UT, shoulder elevator), the posterior deltoid (PD, shoulder extensor), the lateral deltoid (LA, shoulder abduction), the anterior deltoid (AD, shoulder flexor), the pectoralis major (PM, arm adduction), the biceps brachii (BB, elbow flexor) and the lateral triceps (LT, elbow extensor). These muscles are chosen because they are surface muscles and their activation describes most of the upper-limb activity for a desired task. Electrodes for each muscle are placed according to SENIAM guidelines (Hermens et al., 1999).

The level of assistance (*K*_*p*_) provided by the robotic arm and the time (*t*) given to the subject for executing the task are computed in the end-effector robot block shown in Figure 2. On the other hand, the amount of support to be provided to the subject elbow is computed in the arm-weight support block (Figure 2).

The patient biomechanical data acquired through the M-IMU, i.e. the orientation of the hand and the upper-limb acceleration, provided by robot, and the muscular signals, recorded by means of the sEMG sensors are used for (i) reconstructing the kinematics of the subject upper-limb, by means of the Augmented Inverse Kinematics (AIK) (Papaleo et al., 2015), (ii) computing performance indicators, (iii) evaluating the level of muscular fatigue. The obtained data are then used to update robot control parameters (i.e., robot stiffness and the execution time) and the amount of arm support (computed on the basis of the muscular fatigue) for accordingly shaping level of assistance and task complexity in the 3D workspace. Moreover, the elbow Cartesian position provided by the AIK is used in the control of the arm-weight support to track the subject's limb during task execution without interfering with its motion.

### 2.2. Closed-loop control of the bio-cooperative robotic platform

#### 2.2.1. Evaluation of the patient's status

The subjects are constantly monitored during the execution of the task and their status is evaluated through the multimodal interface described in section 2.1. In particular, sEMG, M-IMU, and robot position/force data are acquired to constantly describe the subject's state and to guarantee a strong and safe human-robot interaction.

sEMG signals are used to compute Dimitrov's Spectral Fatigue Index (DI), defined as

(1) $$DI=\frac{\int_{f_{1}}^{f_{2}}f^{-1}*PS(f)*df}{\int_{f_{1}}^{f_{2}}f^{5}*PS(f)*df}$$

where *PS*(*f*) is the signal power spectrum and *f*_1_ and *f*_2_ are the lowest and the highest frequency of the bandwidth. The DI index is computed only during the contraction phase of each muscle. The DI index has been chosen since the literature shows that it is an effective indicator of muscular fatigue and increases with the muscular fatigue (Dimitrov et al., 2006; González-Izal et al., 2012). This parameter, normalized with respect to its maximum value, is estimated for each muscle and then weighted as follows

(2) $$C_{m}=\frac{1}{4}(\frac{1}{4}DI_{BB}+\frac{1}{4}DI_{LT}+\frac{3}{4}DI_{AD}+\frac{3}{4}DI_{LA}+\frac{1}{2}DI_{PD}+DI_{PM}+\frac{1}{2}DI_{UT})$$

Weights were selected through a “trial and error” approach, depending on the contributes of each muscle to the chosen 3D movement. The *C*_*m*_ parameter continuously varies in the range [0, 1]; a threshold strategy is used to evaluate the fatigue level and correspondingly adapt the arm-gravity support level (*L*_*s*_) as

(3) $$L_{s}=\left\{ \begin{array}{l} 0\text{ if }C_{m}<0.20,\\ 1\text{ if }0.20\leq C_{m}<0.40,\\ 2\text{ if }0.40\leq C_{m}<0.60,\\ 3\text{ if }0.60\leq C_{m}<0.80,\\ 4\text{ if }0.80\leq C_{m}<1. \end{array}\right.$$

The so-obtained *L*_*s*_ values correspond to the following values of *K* (Equation 8): 0, 0.25, 0.50, 0.75, 1.

M-IMU and position/force data are acquired at 100Hz and use to reconstruct the subject's arm movement and evaluate biomechanical indicators in order to adapt robot stiffness, as described in section 2.2.3. More in detail, biomechanical indicators, used to describe subject limb movements are (Papaleo et al., 2013):

- *Aiming* *angle* (α) : angle between the desired direction $\vec{tg_{dir}}$ and the real direction of the task from the starting point up to peak speed point $\vec{m_{dir}}$
  (4) $$\alpha =\frac{acos\left(\vec{tg_{dir}}*\vec{m_{dir}}\right)}{\left(\left\|\vec{tg_{dir}}\right\|*\left\|\vec{m_{dir}}\right\|\right)}$$
- *Mean*−*Arrest*−*Period*−*Ratio* (*MAPR*): it represents the ratio between the number of samples (*t*_*perc*_) in which the joint velocity is more than 10% of the peak velocity and the whole task duration (*t*_*tot*_)
  (5) $$\begin{array}{r} MAPR=\frac{t_{perc}}{t_{tot}} \end{array}$$
- *Inter*−*joint* *coordination* (*q*_*corri, j*_): it represents a coordination index beetween two upper-limb joint angles *q*_*i*_ and *q*_*j*_
  (6) $$\begin{array}{r} q_{corri,j}=\frac{R\left(q_{i},q_{j}\right)}{\sqrt{R_{qi}\left(q_{i}\right)*R_{qj}\left(q_{j}\right)}}, \end{array}$$
  where *R*(*q*_*i*_, *q*_*j*_), *R*_*qi*_(*q*_*i*_) and *R*_*qj*_(*q*_*j*_) are covariance and autocovariance matrices
- *Useful*−*Mean*−*Force* *UMF*: it is the mean force along the desired direction $\vec{tg_{dir}}$
- *Useful*−*Peak*−*Force* *UPF*: it is the peak force along the desired direction.

#### 2.2.2. Control of the arm-weight support

In the proposed robotic platform, as shown in Figure 2, arm-weight support allows supporting subject limb based on his/her muscular fatigue. To this purpose, a proportional-derivative (PD) torque control with gravity compensation has been developed in C++ (by using Microsoft Visual Studio Community 2017®). The appropriate torque, to be supplied to the subject for supporting the arm in the correct position, is defined at each iteration as

(7) $$\begin{array}{r} \tau \left(q\right)=\tau_{PD}\left(q\right)+\tau_{g}\left(q\right) \end{array}$$

where τ_*PD*_(*q*) is the PD output torque and τ_*g*_(*q*) is the necessary gravitational torque. The τ_*g*_(*q*) is computed as

(8) $$\begin{array}{r} \tau_{g}\left(q\right)=K\tau_{max}\cos\left(q_{d}-q\right)=K\tau_{max}\cos\left(e\right) \end{array}$$

where K is a constant which ranges between [0, 1], determined according to the patient muscular fatigue (as detailed in section 2.2.1), τ_*max*_ is the maximum torque needed to sustain the subject arm measured through the motor at the beginning of the task, *q*_*d*_ and *q* are crankshaft desired and real position and *e* is crankshaft position error (*q*_*d*_−*q*), respectively. The desired position for the motor (*q*_*d*_) is based on elbow position and is computed as

(9) $$\begin{array}{r} q_{d}\left(t\right)=\frac{g_{ratio}\delta_{cable}\left(t\right)}{\pi \sigma d} \end{array}$$

where *g*_*ratio*_ is the gear ratio of the motor, σ is the encoder dimensionless resolution and *d* is the diameter of the driven pulley linked to motor. In our case, *g*_*ratio*_ = 74, σ = 5*10^−4^ and *d* = 0.14*m*. Let us define the difference between the new cable length and the reference position as

(10) $$\begin{array}{r} \delta_{cable}\left(t\right)=\vec{e}\left(t\right)-\vec{p}. \end{array}$$

where $\vec{e}\left(t\right)$ is the 3D elbow position provided by AIK and $\vec{p}$ is the 3D pulley position in the robot frame. The AIK algorithm is applied to the hand position provided by the robot sensors and to the M-IMU data in order to solve human arm redundancy and compute upper limb joint angles. In particular, the reconstructed elbow position permits to decide if the cable needs to be reeled in or else unrolled according to the patient limb configuration. In brief, the elbow joint Cartesian coordinates are reconstructed as

(11) $$\vec{e}=\left[\begin{array}{c} l_{u}\sin q_{1}\cos q_{2}\\ -l_{u}\cos q_{1}\cos q_{2}\\ -l_{u}\sin q_{2} \end{array}\right]$$

where *l*_*u*_ is the upper-arm length, *q*_1_ and *q*_2_ are the reconstructed shoulder flexion-extension and intra-extra rotation angles. The M-IMU positioned on the subject upper-arm allows determining the *y* elbow component as

(12) $$\begin{array}{r} e_{y}=\frac{-\ddot{a_{y}}l_{u}}{g}=-l_{u}\cos q_{1}\cos q_{2} \end{array}$$

where *g* is the gravity acceleration and $\ddot{a_{y}}$ is the acceleration component along y-axis read by M-IMU sensor.

#### 2.2.3. Control of the end-effector robot

As described in section 2.1, the subject wrist is attached to the robot arm end-effector that provides the user with assistance-as-needed during the execution of a predefined task. The end-effector robot performs a minimum-jerk trajectory with different task durations *t* (i.e., 5, 7.5, 10*s*), defined as follows

(13) $$s=\left\|p_{f}-p_{i}\right\|\;[10{\left(\frac{t_{j}}{t}\right)}^{3}-15{\left(\frac{t_{j}}{t}\right)}^{4}+6({\left(\frac{t_{j}}{t}\right)}^{5}]$$

where *p*_*i*_ is the initial position, *p*_*f*_ is the final position, *t*_*j*_ is the current time value and *t* is the task duration tuned according to Equations (21, 22, and 23). The robot is controlled with an impedance control with a variable stiffness *K*_*r*_ in order to provide three levels of assistance, that correspond to three values of stiffness *K*_*r*_ (i.e., 0.1, 300, 1,000 N/m), and it is able to change task duration (Papaleo et al., 2013), according to

(14) $$\begin{array}{r} {\vec{\tau }}_{cmd}=J^{T}\left(\vec{FT_{c}}\right)+{\vec{f}}_{dynamics} \end{array}$$

where ${\vec{\tau }}_{cmd}$ is the vector of the command torque, *J*^*T*^ is the transposed Jacobian matrix, $\vec{FT_{c}}$ is the vector of Cartesian force, along axes *x*, *y*, *z*, and torques, about axes *z*, *y*, *x*, (i.e. $\vec{FT_{c}}=\left[F_{c,x}\;F_{c,y}\;F_{c,z}\;T_{c,z}\;T_{c,y}\;T_{c,x}\right]$) while ${\vec{f}}_{dynamics}$ is the dynamic model of the robotic arm. $\vec{FT_{c}}$ is computed as

(15) $$F_{c,y}=\{\begin{array}{ll} -k(y-y_{m,j})-d\dot{y}, & y<y_{m,j}\\ 0, & y_{m,j}\leq y<y_{f}\text{ and }y\geq y_{prev}\\ -k(y-y_{prev})-d\dot{y}, & y_{m,j}\leq y<y_{f}\text{ and }y<y_{prev}\\ -k(y-y_{f})-d\dot{y}, & y>y_{f} \end{array}$$

(16) $$\begin{array}{r} F_{c,z}=\{\begin{array}{cc} -k\left(z-z_{m,j}\right)-dż,\; & z<z_{m,j}\\ 0,\; & z_{m,j}\leq z<z_{f}\text{and}z\geq z_{prev}\\ -k\left(z-z_{prev}\right)-dż,\; & z_{m,j}\leq z<z_{f}\text{and}z<z_{prev}\\ -k\left(z-z_{f}\right)-dż,\; & z>z_{f} \end{array} \end{array}$$

(17) $$F_{c,z}=\{\begin{array}{ll} -k(z-z_{m,j})-d\dot{z}, & z<z_{m,j}\\ 0, & z_{m,j}\leq z<z_{f}\text{ and }z\geq z_{prev}\\ -k(z-z_{prev})-d\dot{z}, & z_{m,j}\leq z<z_{f}\text{ and }z<z_{prev}\\ -k(z-z_{f})-d\dot{z}, & z>z_{f} \end{array}$$

(18) $$\begin{array}{r} T_{c,z}=-k_{0}\left(\varphi -\varphi_{m}\right)-d\overset{{}^{\circ}}{\varphi } \end{array}$$

(19) $$\begin{array}{r} T_{c,y}=-k_{0}\left(\theta -\theta_{m}\right)-d\overset{{}^{\circ}}{\theta } \end{array}$$

(20) $$\begin{array}{r} T_{c,x}=-k_{0}\left(\psi -\psi_{m}\right)-d\overset{{}^{\circ}}{\psi } \end{array}$$

where *K*_*r*_ is the robot stiffness, *x*_*m, j*_, *y*_*m, j*_, and *z*_*m, j*_ are the desired positions, computed as reported in Equation (13), *x*_*prev*_, *y*_*prev*_ and *z*_*prev*_ are the previous positions at time *t*_*j*_, *k*_0_ is the Cartesian stiffness for the orientation, *d* is the controller Cartesian damping (constant), φ, θ and ψ are the RPY (Roll-Pitch-Yaw) angles representing the orientation of the end-effector.

The robot stiffness *K*_*r*_ and the task duration *t* are modified according to a threshold strategy based on two parameters, *C*_*kr*_ and *C*_*t*_, evaluated on the basis of the previously described biomechanical indicators as

(21) $$\begin{array}{r} C_{k_{r}}=\frac{1}{2}\alpha +\frac{1}{8}q_{corr1,4}+\frac{1}{8}q_{corr2,4}+\frac{1}{8}UMF+\frac{1}{8}UPF \end{array}$$

(22) $$\begin{array}{r} C_{t}=\frac{1}{2}MAPR+\frac{1}{8}q_{corr1,4}+\frac{1}{8}q_{corr2,4}+\frac{1}{8}UMF+\frac{1}{8}UPF \end{array}$$

The correct level of assistance provided by the robot is estimated as

(23) $$L_{i}=\left\{ \begin{array}{l} 1,\text{ if }0\leq C_{i}<0.52,\\ 2,\text{ if }0.5\leq C_{i}<0.753,\\ 3,\text{ if }0.75\leq C_{i}<1 \end{array}\right.$$

where *i* = *K*_*r*_, *t*. Values of *L*_*i*_ and *L*_*t*_ are used to select the corresponding robot stiffness (0.1, 300, 1,000 N/m) and task duration (5, 7.5, 10*s*).

### 2.3. Experimental setup and protocol

**Figure 3:**
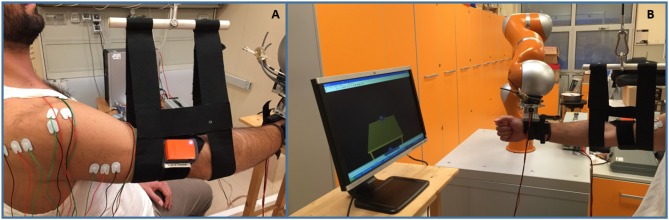
The proposed 3D bio-cooperative robotic platform. **(A)** Detail of M-IMU and sEMG sensors used with arm-weight support; **(B)** Arm-weight support with the whole platform: subject interacts with robotic arm and arm-gravity support.

The proposed robotic platform, shown in Figure 3, is composed of the anthropomorphic robotic arm and the actuated arm-weight support. The robotic arm is the Kuka Light Weight Robot 4+. It is characterized by 7 active Degrees of Freedom (DoFs) and embeds position and torque sensors at joints. The communication between the robot and a remote PC is guaranteed by the Fast Research Interface (FRI) Library. The arm-weight support actuation system is composed of: EC-max 40 brushless Maxon Motor, planetary gearhead Maxon GP 42-C 74:1, Maxon HEDL-5540 encoder and Maxon EPOS2 50/5 control unit. An aluminum pulley, for enveloping the steel rope, (diameter d = 0.14 m) is built-in with motor shaft. Finally, an ergonomic brace for arm support enables to set the correct fitting depending on patients requirements.

Subject upper limb kinematics is reconstructed by means of a Xsens MTw M-IMU sensor.The M-IMU and robot sensors data are acquired at 100 Hz and sent to the AIK algorithm via UDP communication.

sEMG data are collected at 1 kHz, digitized and then filtered by using a sixth-order Butterworth bandpass filter with cutoff frequencies (30,450) Hz and a second-order Butterworth notch filter (50 Hz) to remove noise from power lines. The filtered sEMG signal is normalized with respect to the Maximum Voluntary Contraction (MVC).

Ten right-handed healthy subjects (mean age: 27.9 ± 2.0) have been recruited to participate in this study. All the subjects were able to lift their right arm against gravity, and presented no musculoskeletal or neurological disorders. They provided written informed consent prior to participating in this study. Each subject seated on a chair in front of a screen projecting the virtual reality, as shown in Figure 3. The sensors embedded in the robot arm reconstruct the subject hand position which is used to move the subject hand avatar reproduced in the virtual reality. The virtual reality reproduces the task to be performed and gives the user a continuous feedback on him/her motion performance (in terms of error between the avatar position and the target).

The proposed bio-cooperative system for 3D upper limb rehabilitation allows performing the tasks in two different conditions: (1) without assistance provided by the end-effector robot and by the arm-gravity support and (2) with assistance-as-needed. In condition (2), the level of assistance is tuned on the subject muscular fatigue and on the biomechanical indicators computed during the trials executed without assistance.

The subjects were asked to perform two consecutive sessions in the two conditions. Condition 1 was always executed before condition 2 in order to evaluate all the indicators introduced in section 2.2.1 and correspondingly adapt the robot arm and the arm-weight support behavior. Before each rehabilitation session, an evaluation session is envisaged. When the approach will be tested on patients with severe upper-limb disabilities who are not able to perform the evaluation session without assistance, the computed parameters will suggest to provide the maximum level of assistance.

Each session was composed of two phases of 56 point-to-point movements. Each movement consisted in reaching a target on the screen and then return to the starting point. Targets were placed in 8 different positions, spaced π/4 *rad* apart from North to North-West direction. The transition from one target to another is performed either when the maximum value of the execution time *t* (established by Equation 22) is reached or when position error between the target and the end-effector position is less than a predefined threshold.

During the whole task execution, data from M-IMU, robot sensors and sEMG activities of 7 shoulder and upper-arm muscles were collected.

### 2.4. Statistical analysis

A statistical analysis based on the Wilcoxon paired-sample test was performed for the comparative analysis between the two considered operative conditions (i.e., with and without assistance-as-needed), after verifying that the data did not belong to a Gaussian distribution. In particular, the statistical analysis was performed for comparing (i) the time taken by the subjects for accomplishing the task, (ii) the biomechanical indicators, and (iii) the muscular fatigue in the two conditions. The significance was achieved for *p* < 0.05.

## 3. Experimental results

Each of the ten healthy subjects involved in this study performed the assigned task in the two different conditions previously described.

**Figure 4:**
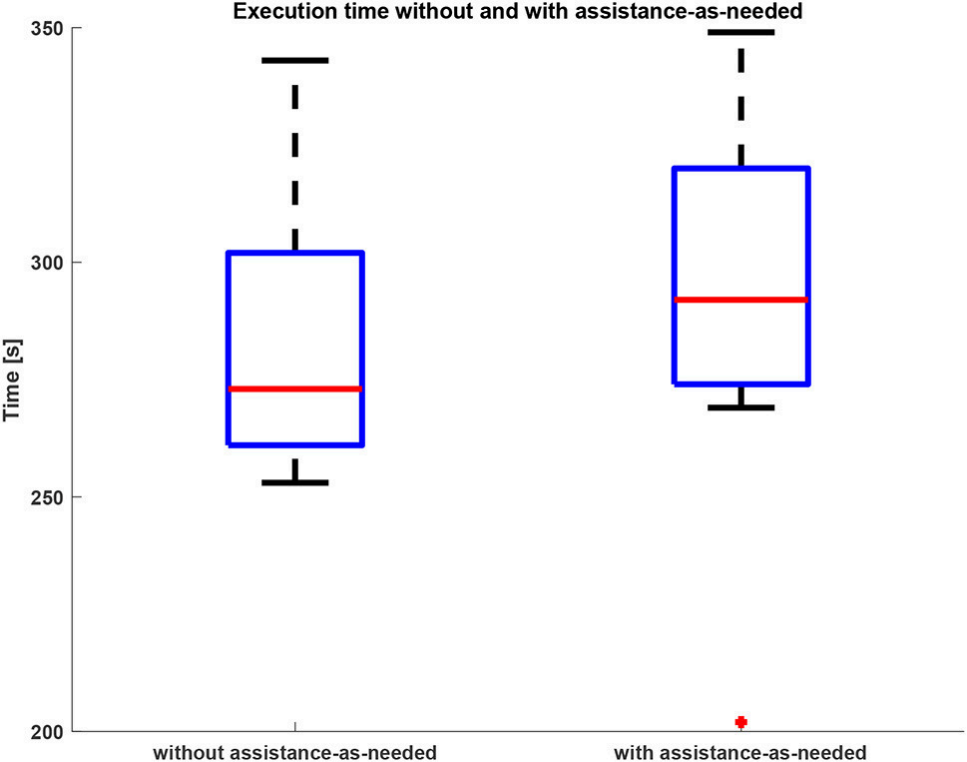
Task duration without and with assistance-as-needed.

The time needed by the subjects for accomplishing the task is reported in the box plots in Figure 4 for both conditions 1 and 2. The subjects performed the assigned task without assistance-as-needed in (283 ± 28)s and with assistance-as-needed in (290 ± 40)s (average times). It was verified that the use of the support does not significantly alter the execution time of the assigned task (Wilcoxon test, *p* = 0.08).

**Figure 5:**
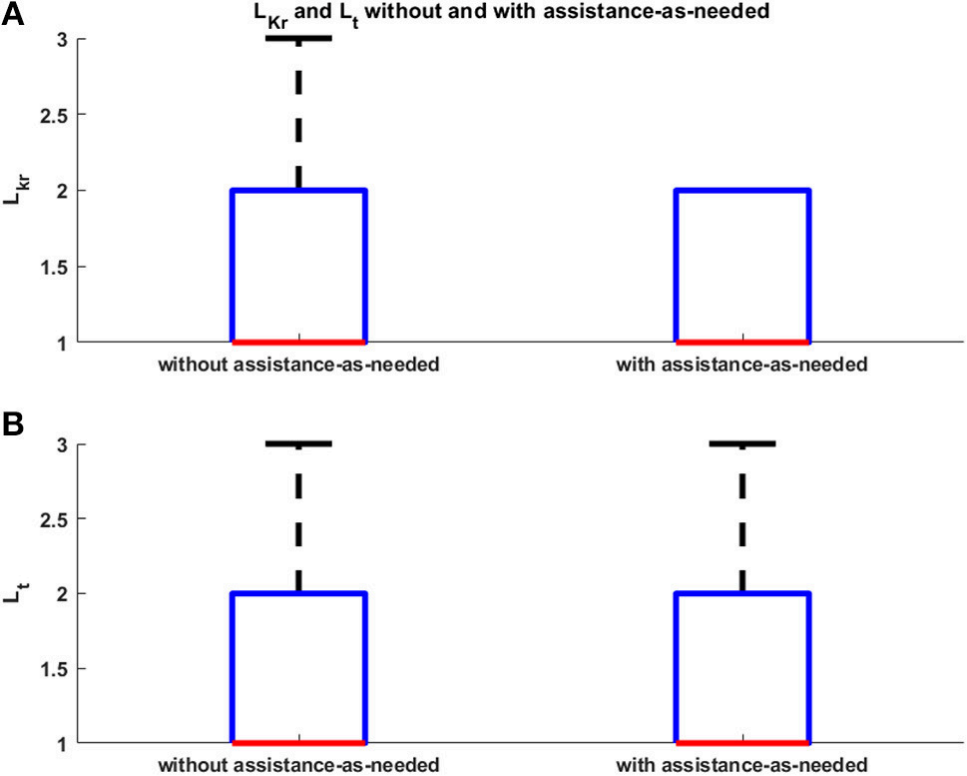
*L*_*kr*_ and *L*_*t*_ without and with assistance-as-needed.

**Figure 6:**
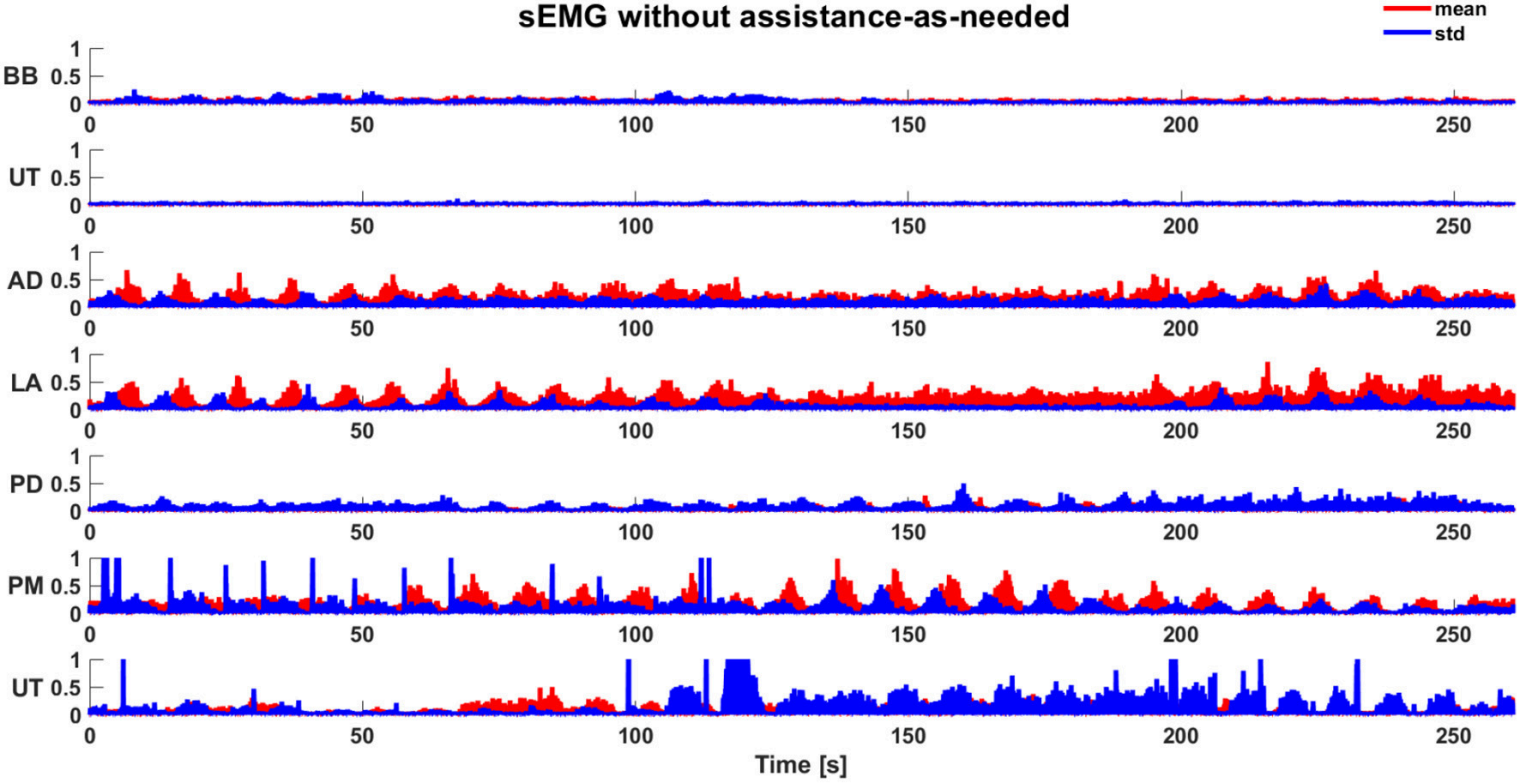
Mean sEMG activity (normalized) and standard deviation during the execution of task without assistance-as-needed.

**Figure 7:**
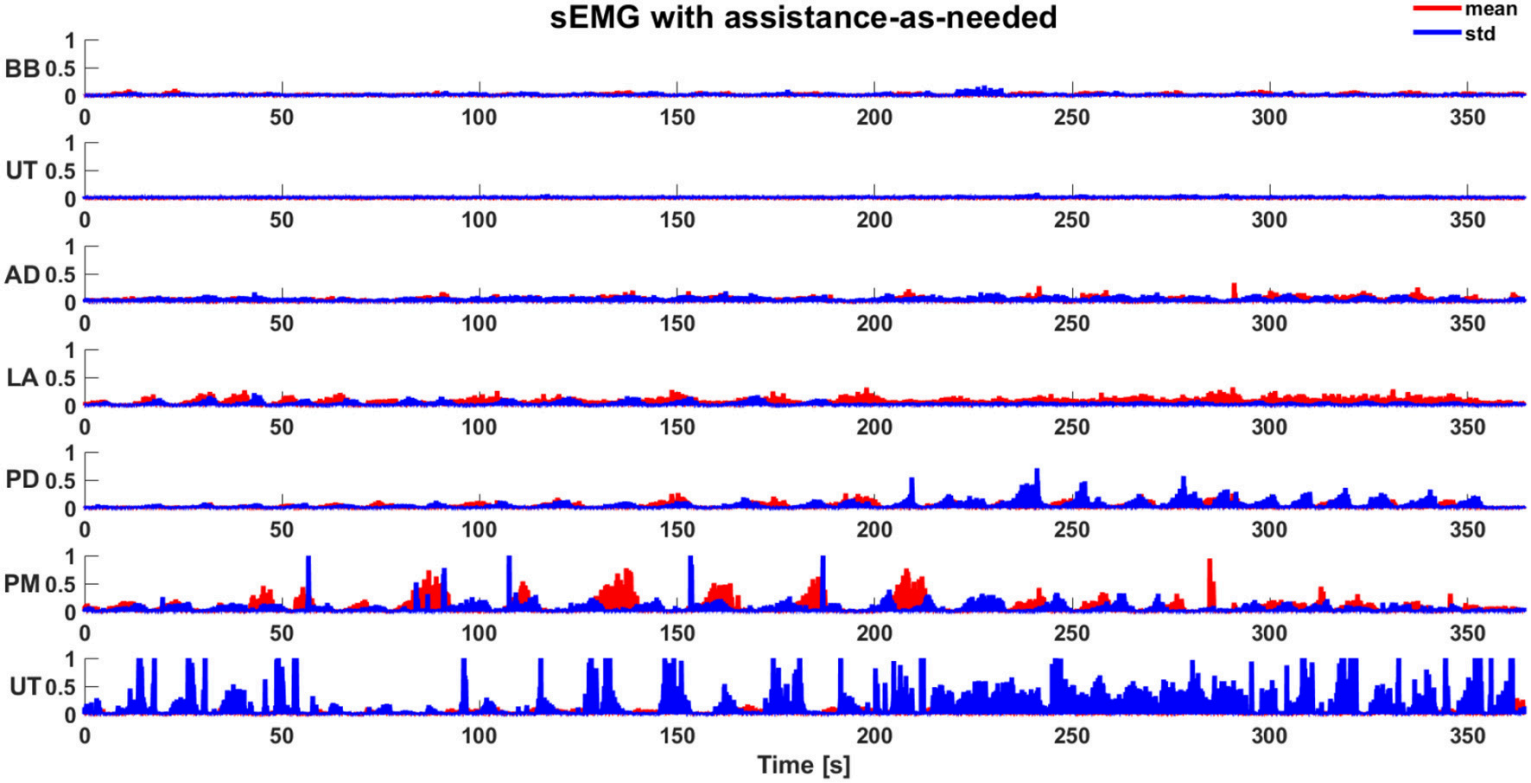
Mean sEMG activity (normalized) and standard deviation during the execution of task with assistance-as-needed.

Robot sensors provided position and force data for customizing the exercises on the basis of subject motor performance. As expected, the computation of the biomechanical indicators for the involved healthy subjects did not show a significant change between the first and the second condition, since they were able to perform the task without any assistance. This is confirmed by the values of the robot stiffness *K*_*r*_ and task duration *t*. The corresponding level of assistance in terms of robot stiffness (i.e., *L*_*kr*_) and time to accomplish the task (i.e., *L*_*t*_) are shown in Figure 5. Indeed, it was demonstrated that, with the proposed system, the biomechanical indicators do not show significant variations due to the introduction of the support (*p* = 0.28 with Wilcoxon test for all the biomechanical indicators evaluated with and without arm-weight support). In Figures 6, 7, the mean EMG activity and its standard deviation, computed on 10 subjects, are reported in both operative conditions, i.e., with and without assistance-as-needed. The 7 sEMG values range between [0, 1] since each of them is normalized with respect to the corresponding MVC. Note that apparently there are not appreciable changes between BB and LT signals, but this is due to normalization with respect to their MVC, so they were activated but their variations are not perceivable.

**Figure 8:**
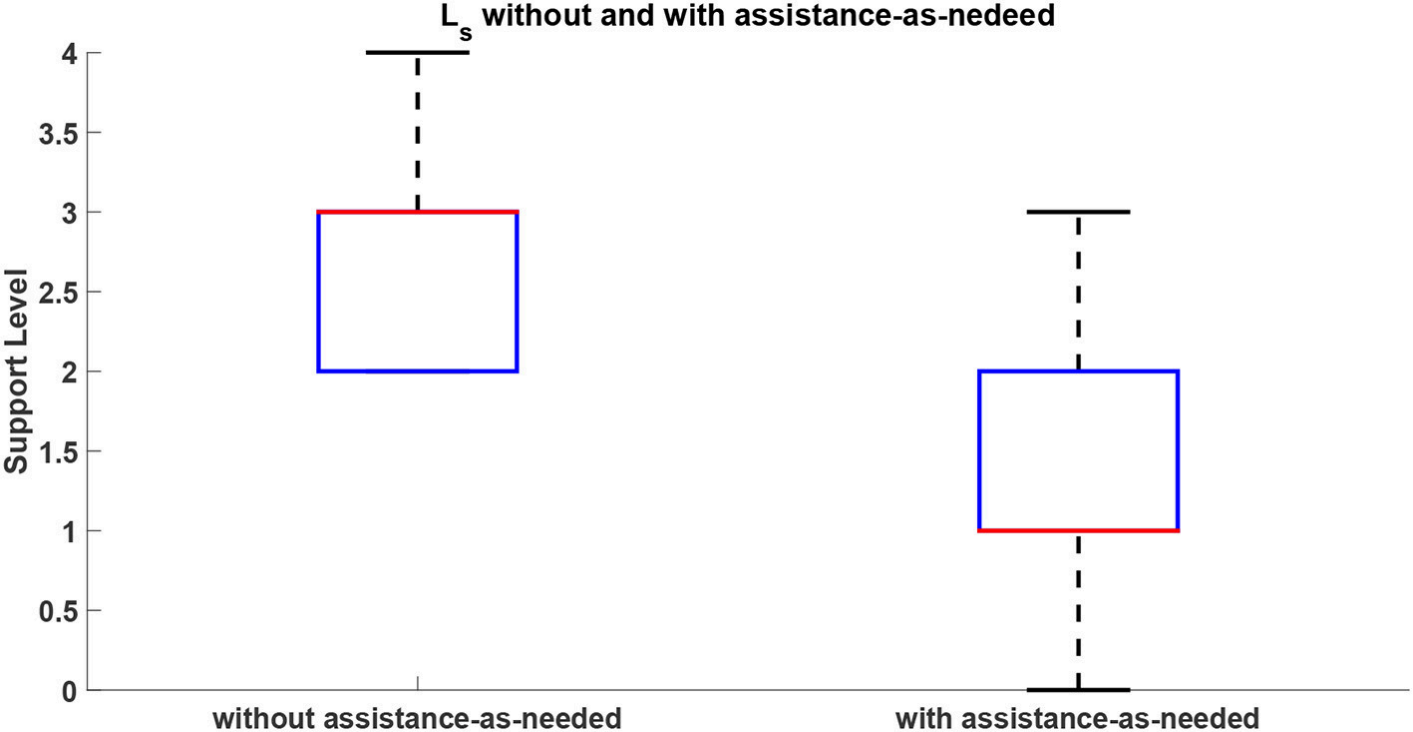
*L*_*s*_ without and with assistance-as-needed.

The EMG signals are used to estimate the level of physical fatigue of the subjects. The corresponding level of arm-weight support is shown in Figure 8 without and with assistance-as-needed. These results show an increase in muscular fatigue emerged during the execution of the task without assistance-as-needed for all the subjects, confirmed by the statistically significant difference in the decrease of fatigue between the two conditions (*p* = 0.03 with Wilcoxon test). The level of support to be applied in Condition 2 is selected on the basis of the fatigue level evaluated during Condition 1, as reported in section 2.2.1. In this way, the support assistance level can be adapted to patient fatigue performance allowing to follow subject arm movements as reported in section 2.2.2.

**Figure 9:**
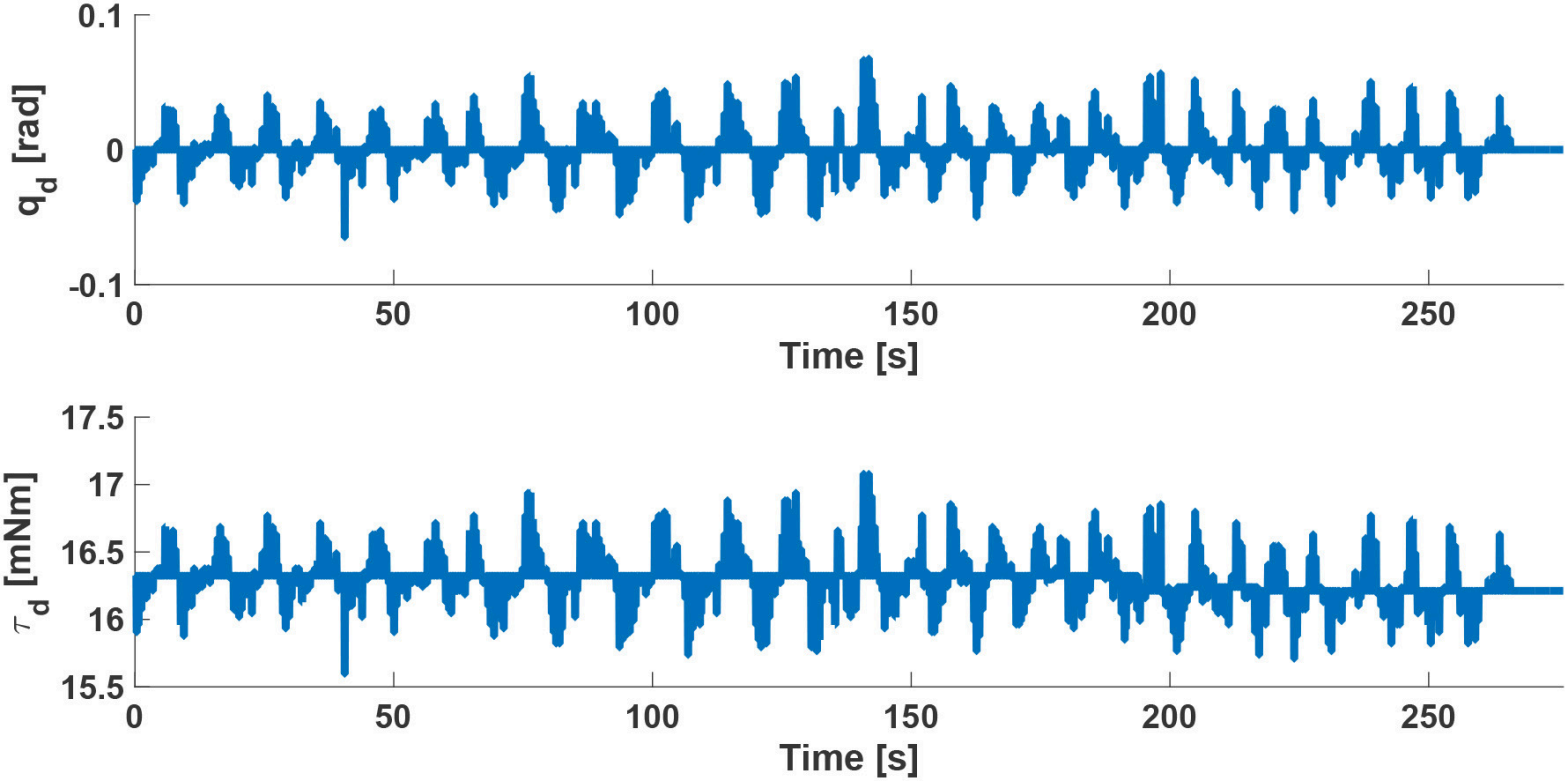
Desired crankshaft position *q*_*d*_ and desired torque τ_*d*_ of the arm-gravity support for a sample subject with a compensation of 50% of the τ_*max*_ necessary to completely support subject arm.

Results about desired crankshaft position (*q*_*d*_) and desired torque (τ_*d*_) are shown in Figure 9 for a sample subject. During the task execution in Condition 1, the level of assistance to be given to the arm support for this subject has been estimated to be equal to the 50% of the τ_*d*_ necessary to completely support subject arm (i.e., τ_*max*_ = 35*mNm*, as evaluated at the beginning of the experimental session).

## 4. Discussions

The movements of the robot end-effector (i.e., subject hand) and elbow position, reconstructed by AIK algorithm, demonstrate that the proposed approach allows executing 3D tasks without interfering with the natural motion pattern and therefore not negatively affecting the motion execution. This is demonstrated by the results of the statistical analysis performed on the biomechanical indicators: their values do not change in a statistically significant manner between the two operative conditions (*p* = 0.45). The reason is that the subjects did not present musculoskeletal or neurological disorders and therefore they were able to perform the assigned task without any assistance. For the same reason, the mean values of task duration confirmed that there is not statistically significant difference between the time obtained without assistance-as-needed and with assistance-as-needed. In fact, the *p* = 0.34 obtained with the Wilcoxon test.

The use of the arm-weight support reduces muscular activity, as evident from Figures 6, 7, also confirmed through Wilcoxon test applied for each muscle (*p* = 0.03). The subjects referred to perceive a reduced muscular fatigue after the introduction of the arm-weight support. This finding could certainly have a huge impact on neuro-rehabilitation. In fact, a reduced muscular fatigue could lead to an increase in therapy session duration and a decrease in wrong arm configurations that may result for compensating for the fatigue of some muscles.

As shown in Figure 9, the control algorithm for arm-weight support allows following subject arm movements and produces a desired torque τ_*d*_, with a profile similar to *q*_*d*_, that is able to both compensate gravity component of the arm and move his/her limb in the 3D space. The proposed strategy, differently from the state-of-the-art, takes into account the relationship between gravity torque of the limb, its dynamics and its dependence on the postures and positions of moving limbs. This platform offers the main advantage, with respect to other platforms in the literature, to provide an adaptable level of both robotic assistance and arm-weight support, thanks to the online computed biomechanical indicators and muscular fatigue, with an expected significant impact on the personalization and optimization of the treatment. Future studies will be conducted to rigorously assess pros and cons of the proposed platform on patient treatment.

The support level applied on the subject arm by the arm-weight support was varied in accordance to the fatigue level estimated for each subject on the basis of Equation (3).

From these results, it is clear that the proposed bio-cooperative robotic platform is based on a closed-loop control that includes the subject, with the aim of executing 3D point-to-point movements adapting to the state of the subject from both biomechanical and muscular fatigue point of views.

## 5. Conclusions

In this paper, a novel 3D bio-cooperative robotic platform based on subject status was presented. Aim of the research was defining and implementing a robot-aided neuro-rehabilitation strategy which includes the patient in the control loop by providing him/her the correct amount of assistance on the basis of biomechanical performance and muscular fatigue indicators. In particular, the interaction between the subject and the proposed platform was constantly monitored to extract biomechanical and muscular indicators and consequently modify the level of assistance and the difficulty of the exercise, in order to demonstrate that the proposed platform does not negatively affect motor execution of the task and muscular activation patterns. The platform was tested on 10 healthy subjects performing a 3D point-to-point movements with and without assistance-as-needed. The obtained results demonstrated that the proposed system reduces the muscular fatigue without negatively influencing correct motor patterns.

Future work will be devoted to extend the study to a higher number of tasks, to test the proposed robotic platform on post-stroke patients, with an *ad hoc* experimental protocol, to establish the effects on patients with motor disabilities.

## Ethics statement

The experimental protocol was approved by the local Ethical committee (Comitato Etico Università Campus Biomedico di Roma, reference number: 01/17 PAR ComEt CBM), by the Italian Ministry of Health (Registro-classif. DGDMF/I.5.i.m.2/2016/1096) and complied with the Declaration of Helsinki. All subjects gave written informed consent in accordance with the Declaration of Helsinki.

## Author contributions

FS analyzed the literature, designed the paper, designed and developed the proposed robotic platform, analyzed the experimental data and wrote the manuscript. DS analyzed the literature, organized the experimental sessions, designed mechanical component of the proposed platform, acquired the data and contributed to the manuscript writing. FC analyzed the literature, contributed to design the control algorithm of the proposed platform, analyzed the experimental data and contributed to the manuscript writing. SM and FD contributed to the design of the experiments and discussed the results. SS discussed the results and supervised the study. LZ contributed to the design of proposed platform, discussed the results, wrote the paper and supervised the study. All the authors read and approved the final version of the manuscript.

### Conflict of interest statement

The authors declare that the research was conducted in the absence of any commercial or financial relationships that could be construed as a potential conflict of interest.
